# The prevalence of vascular and metabolic complications after lung transplant in people with cystic fibrosis in a Dutch cohort

**DOI:** 10.1016/j.clinsp.2023.100274

**Published:** 2023-08-17

**Authors:** Lisa M. Oppelaar, Bart Luijk, Harry G.M. Heijerman, Harold W. De Valk, Gerdien Belle- van Meerkerk

**Affiliations:** Leiden University Medical Center, Leiden, Netherlands

**Keywords:** Cystic fibrosis, Lung transplant, Vascular disease, Metabolic disease, Diabetes, Renal insufficiency

## Abstract

**Background:**

With improvements in care for people with Cystic Fibrosis (pwCF), total survival after Lung Transplantation (LTx) will be longer. Therefore, this population's up-to-date analysis of late-onset post-transplant metabolic and vascular complications will be more relevant in current clinical practice.

**Methods:**

We studied 100 pwCF who underwent an LTx between 2001 and 2020 at the University Medical Centre Utrecht, the Netherlands. The median age at transplant was 31 years and 55 percent was male. We assessed survival, the prevalence of metabolic complications (diabetes, renal damage, dyslipidemia, and metabolic syndrome), and vascular complications (hypertension, heart rhythm disease, micro-, and macrovascular disease). In addition, differences in risks for developing complications based on sex and overall survival were analyzed.

**Results:**

The prevalence of macrovascular disease raised to 15.9 percent 15 years post-LTx. The prevalence of diabetes increased from 63 percent at LTx to over 90 percent 15 years post-LTx and the prevalence of dyslipidemia increased from 21 percent to over 80 percent. Survival 1-, 2-, 5-, and 10 years post-transplant were 84, 80, 76, and 58 percent respectively. No significant differences were found based on sex.

**Conclusion:**

This study shows that the prevalence of cardiovascular risk factors increases after LTx for CF, potentially leading to major complications. These data emphasize the necessity of regular check-ups for metabolic and vascular complications after LTx with specific attention to renal damage. Early recognition of these complications is crucial and will lead to earlier intervention, which could lead to improved prognosis after lung transplantation.

## Highlights

• Cystic fibrosis related vascular complications.
• Survival of people with cystic fibrosis.
• Cystic fibrosis related vascular complications and metabolic complications.

## Background

Cystic Fibrosis (CF) is a severe, recessive hereditary disease mostly affecting the Caucasian population.^1^ Worldwide there are 70.000 people known with CF, most likely even more, with underreporting from less developed regions.^2^ Today, the median survival of people with CF (pwCF) in Europe lies around 30 years.^3^,^4^ The predicted median survival of a person with CF born today is past the age of 40.^4^,^5^ So, the prognosis for pwCF is vastly improving, especially with the introduction of CFTR modulators over the last number of years. The number of pwCF being listed for a lung transplant has fallen and so it is timely and relevant to know the prevalence of cardiovascular and metabolic complications of this patient group post-lung transplant but also pre-lung transplant while they are still followed by their CF physicians as these patients may need a lung transplant in the future. Knowing and treating these complications would be very relevant as it could be a barrier in the future for listing.^3^,^5^

In pwCF, the most affected organ system is the respiratory system. Viscous, adherent secretions cause poor airway clearance and continuous infections.^6^ These infections come with severe inflammation, causing bronchiectasis and lung damage. In the end, respiratory failure is most likely to occur, which is the leading cause of death in pwCF. Up to 73% of all CF deaths are respiratory-related.^3^, ^4^, ^5^ The only therapy for end-stage CF lung disease is an LTx. The 1-, 3- and 10-year survival for pwCF after LTx are 93.6%, 71.4%, and 53.6% respectively.^7^, ^8^, ^9^ Mortality after LTx in pwCF is mainly due to chronic lung allograft dysfunction or sepsis.^9^

Besides lung damage, an often-occurring complication of CF is exocrine pancreatic insufficiency, which could lead to CFRD. Of all pwCF, 65% to 96% use pancreatic enzyme replacement therapy.^3^,^4^,^10^ The overall prevalence of increases with age and is higher than 30 percent in patients older than 30 years.^4^,^11^ Tacrolimus and prednisone can also cause diabetes, making the risk of developing New-Onset Diabetes After LTx (NODAT) even bigger.^12^

Microvascular complications, such as neuropathy, nephropathy, and retinopathy, are some of the complications described in CFRD patients. These complications are more common in CFRD patients with fasting hyperglycemia, longer CFRD duration, and poorer control of the disease.^13^,^14^ Nephropathy can also be caused by nephrotoxic drug use, such as tacrolimus which is given after LTx.

As the general prognosis in pwCF continues to improve, more problems from long-term CF complications such as CFRD and the need for lung transplants will be present. Also, more patients will be transplanted at a later age and live longer after LTx. Up-to-date analysis on pwCF, specifically after transplant, regarding vascular and metabolic complications could help get insight and raise awareness for the potential benefit of screening and treating those complications early. The aim of this study, therefore, is to map those vascular and metabolic complications of pwCF before and after LTx. There will also be looked at the influence of sex for developing certain complications and overall survival. The main research questions of this study are as follows: *What is the prevalence of metabolic and cardiovascular complications after LTx in the CF population? Does sex influence the risk for these complications? And lastly, what is the survival post-lung transplant?*

## Methods

### Subjects

For this retrospective cohort study, all pwCF who underwent an LTx between 2001 and 2020 at the University Medical Center Utrecht, the Netherlands were included. Data were gathered from electronic hospital charts and data from clinical care and all data were pseudonymized before analysis. Follow-up was centered when patients were referred to another hospital, in case of retransplantation or death. Otherwise, data were collected until December 2020. Because the study was retrospective and did not subject the participants to any actions, this study was waived by the local medical ethics committee. As the quantity of the cohort was large and some participants had already passed away, no informed consent was necessary. However, if patients had ever declared that their data could not be used for any research purposes, they were excluded (n = 4).

### LTx protocol

Before an LTx, pwCF had a workup including lab analysis and an ECG. Micro- and macrovascular disease was only investigated when clinical symptoms were present.

After an LTx a standard immunosuppressive regime was started consisting of prednisone, tacrolimus, and mycophenolate motefil. After the transplant, prednisone was slowly tapered to 10 mg per day for chronic use. The amount of tacrolimus given was measured by blood levels. The first year after the transplant consisted of several check-ups. After 1-year this was reduced to a check-up every 3-months. During these check-ups, blood pressure was measured, and regular laboratory blood tests were performed, including renal function and random glucose level. Also, annually plasma glucose and lipids were checked in a fasting state. Patients with CFRD or NODAT were annually tested for microvascular complications. Since 2008, a 75 gram oral glucose tolerance test was done annually for every non-diabetic patient.

### Diabetes

The diagnostic criteria for diabetes were done according to the World Health Organization and the international diabetes federation.^15^ Diabetes was diagnosed if fasting plasma glucose > 7.0 mmoL/L or 2h plasma glucose > 11.1 mmoL/L on at least two separate days. So, every patient with one divergent lab result was tested later again to either confirm or negate the diagnosis at that time. Besides that, the use of glucose-lowering drugs was diagnostic for diabetes. Glycated hemoglobin was not analyzed, because this marker has less value in pwCF. This is due to their chronic inflammation.

### Chronic renal damage/kidney insufficiency

Renal Insufficiency (KI) was defined as the estimated Glomerular Filtration Rate (eGFR) < 60 mL/min/1.73 m^2^ or if the patient had micro- or macroalbuminuria. If the decrease of eGFR was only temporary (< 3-months) this was not counted as KI.^16^

### Dyslipidemia

The definition of dyslipidemia was based on the international guideline of the American College of Cardiology and the American Heart Association.^17^ At least one of the following criteria on two separate days was required for diagnosing dyslipidemia: low-density lipoproteins > 2.5 mmoL/L or high-density lipoproteins < 1.0 mmoL/L in males or < 1.3 mmoL/L in females or triglycerides ≥ 1.7 mmoL/L. All measurements were done in the fasting state. If a patient used lipid-lowering medication this was also diagnostic for dyslipidemia.

Waist circumference is also an important indicator of dyslipidemia but is not taken into account in this study as those data were not available.

### Metabolic syndrome

According to the international joint scientific statement on harmonizing the Metabolic Syndrome (MS)^18^ at least three out of the following five risk factors have to be present in the patient for diagnosis of metabolic syndrome: hyperglycemia (glucose ≥ 11.1 mmoL/L), elevated triglycerides (≥ 1.7 mmoL/L), overweight (Body Mass Index ‒ BMI ≥ 25.0 kg/m^2^), reduced high-density lipoproteins (< 1.0 mmoL/L [males] or < 1.3 mmoL/L [females]) and elevated blood pressure (> 140 mmHg systolic or > 90 mmHg diastolic).

### Hypertension

Hypertension was diagnosed according to the 2014 evidence-based guideline for the management of high blood pressure in adults.^19^ If a patient had a systolic blood pressure > 140 mmHg or diastolic blood pressure > 90 mmHg in at least two consecutive measurements at least 24 hours apart, this was diagnosed as hypertension. If a patient used blood pressure-lowering medication this was also diagnostic for hypertension.

### Microvascular disease

The microvascular disease was diagnosed through clinical care. Retinopathy, neuropathy, and nephropathy were studied. Retinopathy was diagnosed by fundoscopy. Neuropathy was tested at physical examination and by medical history. Micro- and macroalbuminuria were measured in a urine sample, with no 24 hour collection. Nephropathy was difficult to determine if it was due to microvascular disease, diabetes, hypertension, nephrotoxic drugs (i.e., tacrolimus), other causes, or a combination of those factors. If it was specified by the clinical practitioner that the nephropathy was caused by nephrotoxicity due to nephrotoxic drugs, this was not counted as microvascular disease.

### Macrovascular disease

The following macrovascular complications were studied: Myocardial Infarction (MI), stroke, Transient Ischemic Attack (TIA), Peripheral Vascular Disease (PVD), and stable angina pectoris. MI was diagnosed with either significantly elevated biomarkers or changes in Electrocardiogram (ECG). A stroke was confirmed with imaging techniques. A TIA was diagnosed through clinical symptoms and with no signs of permanent damage or infarction by a neurologist. PVD was confirmed with ankle-arm index (< 0.9) or another equally reliable technique. At last, stable angina pectoris was confirmed with clinical symptoms of transient exercise-induced and confirmed by an abnormal exercise stress test.

### Heart rhythm disease

All arrhythmias confirmed with ECG were diagnosed as Heart Rhythm Disease (HRD). If an arrhythmia was only temporary (< 1-month) this was not counted as HRD.

### Statistical analysis

All statistical analyses were done in SPSS version 26.

Differences in the overall prevalence of complications and mean BMI before versus after LTx were tested with repeated measurements of Analysis of Variance (ANOVA).

Differences between the prevalence of complications in male and female subjects at baseline were tested with the 2-sided Chi-Square test.

Hazard ratios with confidence intervals and p-values of the prevalence of complications in male and female subjects were measured with the Cox proportional hazards model.

Differences in mean BMI and mean age were measured with the Mann-Whitney *U* test. *t*-tests were not compatible, as the BMI and age were not normally divided.

Mann-Whitney *U* tests were also used for measuring the possible effect of year of LTx on survival. For measuring the effect of sex, age at LTx, and having CFRD before LTx on survival, cox proportional-hazard models were used.

## Results

### Subjects

For the present study, we included 100 pwCF that underwent an LTx. The median age at transplant was 31 years and 55 percent was male. Characteristics of male and female subjects were compared at baseline (Table 1). Microvascular complications were not routinely investigated before LTx, so the prevalence could be underestimated (1.1%). No significant differences were found in mean BMI or prevalence in vascular or metabolic complications. Males showed to be significantly older than females in this cohort (*p* = 0.047).

**Table 1:** Characteristics of the cohort at baseline (before LTx).

	Number of subjects available for analysis			Total cohort a	Male	Female	p-value
	Male	Female	Total		n (%)	n (%)	
Hypertension	55	45	100	4 (4%)	2 (3.6%)	2 (4.4%)	1.0
Cystic fibrosis-related diabetes	55	45	100	63 (63%)	32 (58.2%)	31 (68.9%)	0.3
Renal insufficiency	55	45	100	3 (3%)	1 (1.8%)	2 (4.4%)	0.6
Dyslipidemia	34	23	57	12 (21.1%)	6 (17.6%)	6 (26.1%)	0.5
Metabolic syndrome	47	38	85	2 (2.4%)	0 (0%)	2 (5.3%)	0.2
Microvascular complications	51	44	95	1 (1.1%)	0 (0%)	1 (2.3%)	0.5
Macrovascular complications	51	43	94	1 (1.1%)	0 (0%)	1 (2.3%)	0.5
Heart rhythm disease	43	37	80	10 (12.5%)	4 (9.3%)	6 (16.2%)	0.5
Median age at LTx	55	45	100	31.0	35.0	28.0	0.047^b^
Median body mass index	53	44	97	19.7	19.7	19.7	0.4

The table shows the characteristics of the cohort at baseline. The p-values show the comparison between male and female subjects at baseline.

a n (%) for specified conditions. For continuous variables the median was shown.

b p-value showed statistical significance: p ≤ 0.05

### Primary outcomes

Prevalence of vascular and metabolic complications before and after LTx

**Fig. 1:**
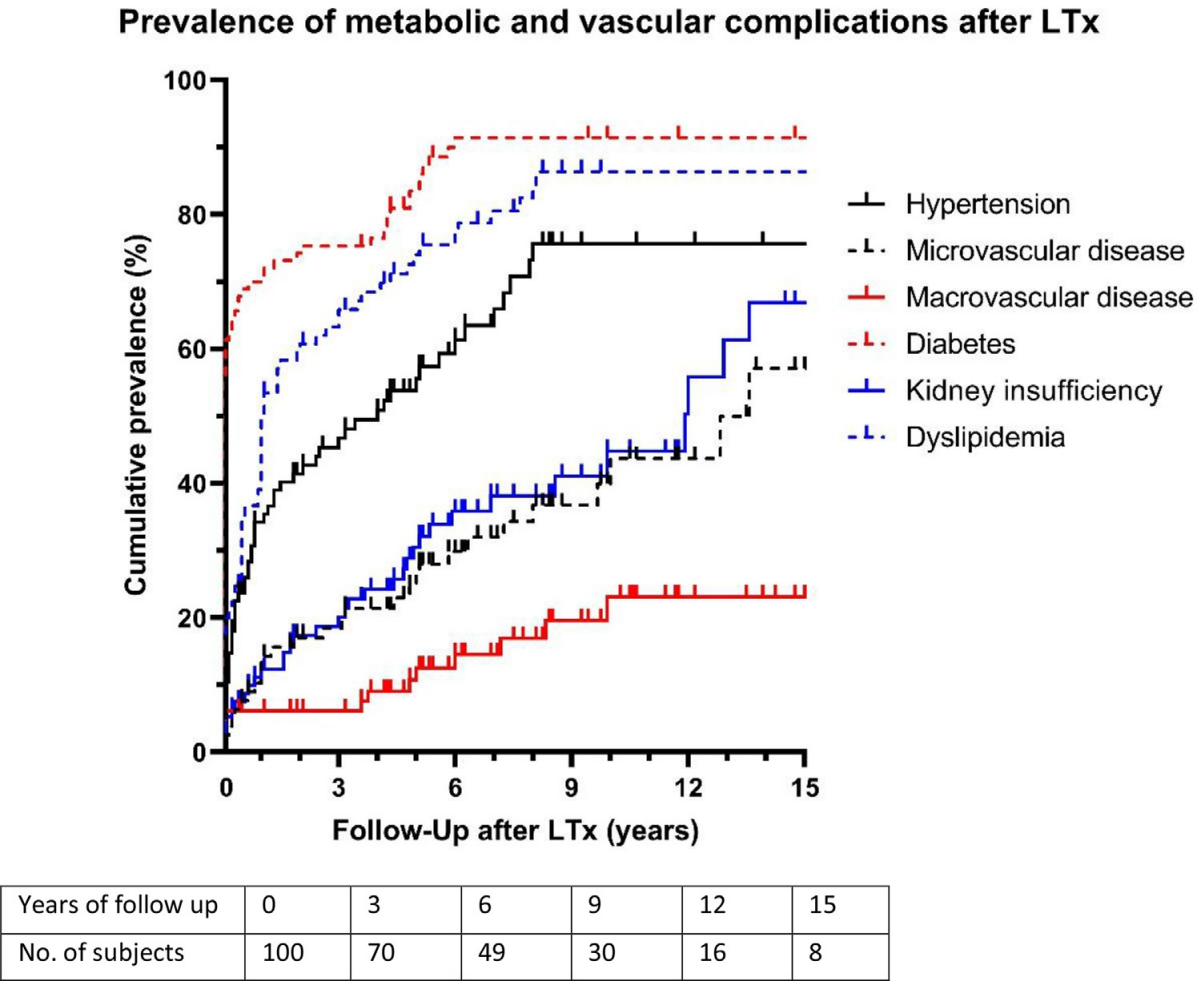
Prevalence of metabolic and vascular complications after LTx over time. The prevalence of metabolic complications after LTx was visualized with maximal follow-up of 15 years. Note: Colors of Figure 1 should be used when printed.

The overall prevalence of metabolic and vascular complications before and after LTx was compared to the prevalence of these complications in the first 15-years after LTx was visualized in Fig. 1. All complications showed an increase over time. Especially diabetes, which was present in 63 percent of the cohort before LTx and in 89 percent, 6-years post-LTx (*p* = 0.001). The prevalence of renal insufficiency kept rising after LTx and is at 65 percent, 15-years post-LTx (*p* ≤ 0.001).

In the table underneath Fig. 1, the number of subjects available for analysis was shown. This number decreases over time as some patients are less than 15-years past their LTx at the time of analysis or are deceased before reaching 15-years past LTx.

## Macrovascular disease

Of the 82 patients that were available for analysis of macrovascular disease, 13 (15.9%) showed macrovascular disease after LTx. Of these 13 patients, 1 (1.2%) had an MI, and 6 (7.3%) had a stroke. Another 6 (7.3%) patients had PVD and 2 (2.4%) suffered from heart failure. Of these 13 patients, there were two who had multiple macrovascular diseases. One had a stroke followed by heart failure and the other had PVD followed by a stroke.

Higher age at LTx showed to have a significant effect on developing macrovascular disease after LTx (HR = 1.06; 95% CI 1.003‒1.12). Also having hypertension before LTx showed to significantly increase the risk of developing macrovascular disease (HR = 8.9; 95% CI 1.8‒44.7).

Differences between male and female subjects regarding vascular and metabolic complications

We investigated if either males or females were more likely to develop certain vascular or metabolic complications (Table 2). As males were significantly older at LTx, hazard ratios were measured stratified for age at LTx. The hazard ratio of females for developing renal insufficiency trended towards significance with an HR of 2.77 (95% CI 0.93‒8.3; *p* = 0.067). No other complications showed a significant difference between males and females.

**Table 2:** Prevalence of complications in male and female subjects during maximal 15 years of follow-up after LTx, stratified for age at LTx

	Number of subjects available for analysis		Male (n = 55)	Female (n = 45)	Hazard Ratio Age-adjusted	95% Confidence Interval Age-adjusted
	Male	Female	n (%)	n (%)		
Hypertension	55	45	30 (54.5%)	26 (57.8%)	1.03	0.5‒2.2
New onset diabetes after LTx	55	45	13 (23.6%)	11 (24.4%)	1.58	0.6‒4.5
Total diabetes after LTx	55	45	46 (83.6%)	43 (95.6%)	1.22	0.7‒2.1
Renal insufficiency	55	43	14 (25.5%)	20 (46.5%)	2.77^a^	0.93‒8.3
Dyslipidemia	47	38	34 (72.3%)	34 (89.5%)	1.43	0.7‒3.0
Metabolic syndrome	46	38	20 (43.5%)	27 (71.1%)	1.86	0.8‒4.2
Microvascular disease	44	35	14 (31.8%)	14 (40.0%)	1.07	0.3‒3.1
Macrovascular disease	44	38	3 (6.8%)	10 (26.3%)	51.92	0.1‒44986
Heart rhythm disease	54	43	13 (24.1%)	14 (32.6%)	0.92	0.3‒2.5

The prevalence of vascular and metabolic complications after LTx was compared between male and female subjects, stratified for age at the time of LTx.

a Trends toward significance.

### Secondary outcomes

Survival

**Fig. 2:**
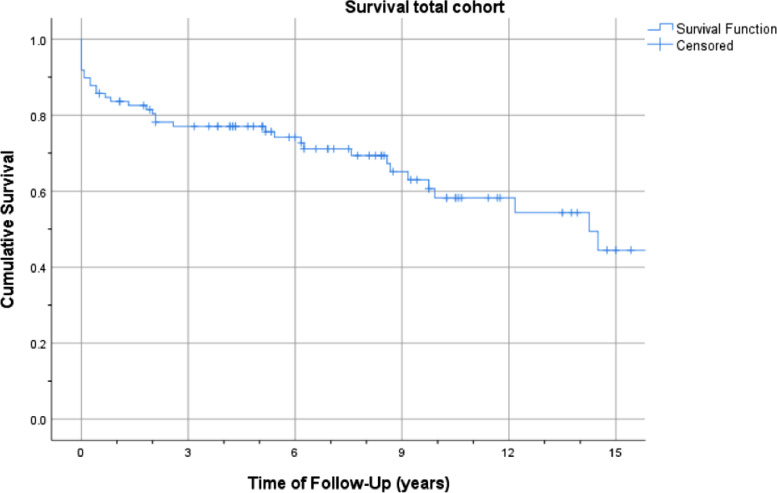
Kaplan-Meier Curve Overall Survival Cohort. The survival of the total cohort was visualized as a Kaplan-Meier curve in graph 3. Survival 1-, 2-, 5-, and 10-years post-transplant were 84, 80, 76, and 58 percent respectively.

The survival of the total cohort was visualized as a Kaplan-Meier curve (Fig. 2). One-, 2-, 5- and 10-year survival post-LTx were 84, 80, 76, and 58 percent respectively. Median survival after LTx was 14-years.

Measuring the effect of sex on survival showed no significance (HR = 1.25; 95% CI 0.6‒2.4). Also, no effect was found on survival for age at LTx (HR = 1.02; 95% CI 0.99‒1.1) and CFRD diagnosis before LTx (HR = 0.94; 95% CI 0.5‒1.9).

Results of microvascular disease in the diabetic subjects

In the diabetic subjects of this cohort, the prevalence of microvascular complications was measured (n = 89). Retinopathy was found in 6.7 percent of the subjects, neuropathy in 14.6 percent, and nephropathy in 10.1 percent.

## Discussion

The present study is the first, to the authors’ knowledge, to investigate the prevalence of metabolic as well as vascular complications following an LTx in a CF cohort. The study showed that the prevalence of cardiovascular risk factors increases after LTx for pwCF. Also, renal insufficiency is an important complication after LTx. Females may be more prone to developing renal insufficiency than males although this did not reach significance in this study

In this study, macrovascular disease was prevalent in 13 out of 82 patients (15.9%). No similar studies were found that studied CF patients after LTx for macrovascular disease. Silverborn et al. researched the new-onset cardiovascular risk factors in LTx patients.^20^ Their results showed a higher prevalence of hypertension, dyslipidemia, and diabetes mellitus after LTx, which is similar to the present study. However, they only looked at the risk factors instead of the macrovascular risk itself. Hence, this study was not comparable.

The ISHLT records and publications show no specific data on macrovascular disease itself. It presents cardiovascular disease as a cause of death. From 1995 to 2018 the prevalence of cardiovascular cause of death in the first 10-years post-LTx was 6.8 percent. Comparing this data to the data is difficult, as the studied population is more specific and younger at transplant. Furthermore, macrovascular disease as a cause of death and macrovascular disease itself are hard to compare.^21^

According to Nash et al. even though risk factors could be present in CF patients, cardiovascular risks seem to remain low in this population.^22^ They included 108 pwCF after LTx and followed their lipids and other cardiovascular risk factors. However, the study does not specify how cardiovascular disease itself was analyzed. They only state that there was no documented clinical evidence of cardiovascular disease in the post-LTx cohort. So, cardiovascular disease was not defined and there was no clear description as to how they collected data on cardiovascular disease. Meaning, the present study brings novel information about the prevalence of macrovascular disease in pwCF after LTx.

The rising prevalence of macrovascular disease after LTx may be explained by increasing cardiovascular risk factors after LTx. Known risk factors for macrovascular disease are dyslipidemia, Metabolic Syndrome (MS), and diabetes mellitus. In this study, the prevalence of dyslipidemia, MS, and diabetes also increased significantly after LTx. With these increments, it would be logical that also the risk for macrovascular disease raised remarkedly. Also, Poore et al. pointed out the possibility that more cardiovascular disease can be seen in the CF population as it grows older. ^23^

Higher age at LTx was found to have a significant effect on developing macrovascular disease as well as hypertension before LTx. However, only 4 patients of the whole cohort had hypertension before LTx and only 1 of them developed macrovascular disease. No other studies were found specific for testing possible risk factors for macrovascular disease in the CF population after LTx.

Higher age is a known risk factor for developing macrovascular disease in the general population.^24^,^25^ We confirmed that in the CF population, this is most likely also applicable. Furthermore, hypertension is also known to be a risk factor for developing macrovascular disease, and in the CF population, this could also be applied.^25^,^27^ Even though the numbers were too low to draw significant conclusions from, this could be a possible explanation as to why having hypertension could be related to higher chances of macrovascular disease in pwCF after LTx. Further research with a larger cohort is necessary to confirm this possible explanation.

In this study, the prevalence of renal insufficiency was almost 20% higher in females than in males with an HR of 2.77 (95% CI 0.93‒8.3; *p* = 0.067). Quon et al. analyzed 993 pwCF after LTx specifically for renal dysfunction.^28^ They came to a similar conclusion that females with CF seemed to have higher chances of developing renal insufficiency with an HR of 1.56 (95% CI 1.22‒1.99). No other studies that researched renal insufficiency were found specific for pwCF after LTx.

Other studies showed no difference in prevalence between male and female subjects but were missing either specification for CF or LTx, which made these studies incomparable.^29^,^30^ This suggests that although female LTx recipients, in general, may not have higher chances of developing renal insufficiency, female recipients specifically with CF possibly do. Females in general have lower nephron numbers than males, which means less reserve of renal function.^31^ Tacrolimus and other calcineurin inhibitors are known for their nephrotoxicity. Doses of tacrolimus after LTx will be adjusted based on serum creatinine or tacrolimus through blood. Although, in general, these factors give a valid indication of the level of toxicity, often sex differences are not taken into account. Females tend to have lower muscle mass, which can result in ‘normal’ creatinine levels even when their kidneys are in distress. So, the most plausible reason for females with CF to have a higher prevalence of renal insufficiency after LTx, we speculate, is the lower nephron mass and the consequences of not monitoring nephrotoxic drugs specifically for sex. More research would be necessary to confirm this speculation.

A strength of the present study was its all-inclusiveness. As the general CF population is fairly diverse, we did not want to only include a specific type of pwCF in the present study. For example, no person was excluded because of the degree of severity of the disease or because of higher age at transplant. So, the outcomes of the study can be translated into the general adult CF population.

As the data is collected from only the University Medical Centre Utrecht, inter-hospital differences are eliminated. Local guidelines and most staff involved remain the same for one hospital. Therefore, fewer variables in care exist in this cohort.

The study is retrospective, and mainly descriptive in character, which could be a weakness because not all data is specifically collected for the study. For example, not all patient data is complete. However, all data were collected from standard care, which is done via a standard protocol. Therefore, most data is complete and collected uniformly. Furthermore, because this study was retrospective and all data was already collected, no actions or consequences were introduced to the participants, which could be seen as a strength. Only microvascular disease was not routinely researched in every patient before the LTx. This could mean that the prevalence of microvascular disease before LTx was underestimated, which could pose a selection bias when comparing before and after the LTx. However, this does not take away the significance of the overall prevalence after the LTx, as patients were yearly checked for microvascular disease after LTx and were at a rather young age at the time of LTx with lower change for cardiovascular complications in previous years without additional immunosuppressive risk for side effects.

We included 100 patients which could be seen as a small-sized cohort. However, in the present study, metabolic complications were researched as well as vascular complications. Moreover, in comparison to other studies that investigated only the prevalence of vascular complications in pwCF, the number of patients is relatively large. To our knowledge, the present study contains the largest cohort for pwCF after LTx investigating the prevalence of vascular complications.

## Conclusion

This study shows that the prevalence of cardiovascular risk factors increases after LTx for pwCF, potentially leading to major complications. These data emphasize the necessity of regular check-ups for metabolic and vascular complications after LTx with specific attention to renal damage, diabetes, and in the diabetic population macrovascular disease. It is necessary to avoid these check-ups falling behind schedule because earlier recognition of these complications leads to earlier treatment, which could lead to an improved prognosis. Further research is needed to confirm if early recognition leads to improved prognosis and fewer complications.

## Declaration of Competing Interest

The authors declare no conflicts of interest.
